# The Nitric Oxide Donor, *S*-Nitrosoglutathione, Rescues Peroxisome Number and Activity Defects in *PEX1G843D* Mild Zellweger Syndrome Fibroblasts

**DOI:** 10.3389/fcell.2021.714710

**Published:** 2021-08-09

**Authors:** Yidi Liu, Ceileigh M. Weaver, Yarina Sen, Gary Eitzen, Andrew J. Simmonds, Lilliana Linchieh, Olivier Lurette, Etienne Hebert-Chatelain, Richard A. Rachubinski, Francesca Di Cara

**Affiliations:** ^1^Department of Cell Biology, University of Alberta, Edmonton, AB, Canada; ^2^Department of Microbiology and Immunology, IWK Research Centre, Dalhousie University, Halifax, NS, Canada; ^3^Department of Biology, University of Moncton, Moncton, NB, Canada

**Keywords:** peroxisome, *PEX1G843D* mutation, Zellweger spectrum disorders, *S*-nitrosoglutathione, high-throughput screen, drug library, fatty acids, *Drosophila melanogaster*

## Abstract

Peroxisome biogenesis disorders (PBDs) are a group of metabolic developmental diseases caused by mutations in one or more genes encoding peroxisomal proteins. Zellweger syndrome spectrum (PBD-ZSS) results from metabolic dysfunction caused by damaged or non-functional peroxisomes and manifests as a multi-organ syndrome with significant morbidity and mortality for which there is no current drug therapy. Mild PBD-ZSS patients can exhibit a more progressive disease course and could benefit from the identification of drugs to improve the quality of life and extend the lifespan of affected individuals. Our study used a high-throughput screen of FDA-approved compounds to identify compounds that improve peroxisome function and biogenesis in human fibroblast cells carrying the mild PBD-ZSS variant, *PEX1G843D*. Our screen identified the nitrogen oxide donor, *S*-nitrosoglutathione (GSNO), as a potential therapeutic for this mild form of PBD-ZSS. Further biochemical characterization showed that GSNO enhances both peroxisome number and function in *PEX1G843D* mutant fibroblasts and leads to increased survival and longer lifespan in an *in vivo* humanized *Drosophila* model carrying the *PEX1G843D* mutation. GSNO is therefore a strong candidate to be translated to clinical trials as a potential therapeutic for mild PBD-ZSS.

## Introduction

Peroxisomes are cellular organelles present in almost every eukaryotic cell and are the site of essential metabolic reactions for organismal development and survival. Matrix enzymes within the peroxisome metabolize reactive oxygen and nitrogen species and catalyze the β-oxidation of very-long chain fatty acids (VLCFAs), synthesis of ether lipids, and processing of other complex lipids (Wanders and Waterham, 2006). Peroxisome biogenesis, fission, and maturation through the import of proteins into the peroxisome matrix are dependent on a set of peroxin (PEX) proteins (Smith and Aitchison, 2013). Mutations in the genes encoding PEX proteins impair peroxisome biogenesis and function, and underlie a clinical spectrum of disorders known as the Peroxisome Biogenesis Disorders (PBDs). PBDs are divided into two distinct syndromes: Zellweger syndrome spectrum (PBD-ZSS) and Rhizomelic Chondrodysplasia Punctata spectrum (PBD-RCDP). With the exception of the *PEX7* mutation underlying most cases of PBD-RCDP, most other *PEX* gene mutations result in generalized peroxisome dysfunction and the PBD-ZSS phenotype (Braverman et al., 2013).

The metabolic dysfunctions that arise in PBD-ZSS often affect the development of multiple organs, including the brain (Braverman et al., 2013). While patients with severe PBD-ZSS can present with refractory epilepsy, neuronal migration disorders, and leukodystrophy, patients with mild cases of PBD-ZSS can exhibit sensorineural hearing loss, retinal abnormalities, leukodystrophy, and developmental and cognitive delays. While severe PBD-ZSS is often detected in the neonate and results in death in the first year of age, patients with mild disease present after the newborn period and can live into adulthood.

The clinical severity of PBDs varies with the genetic basis of the disease. Pathogenic variants in the *PEX1* gene are the most common cause of PBD-ZSS and are found in almost 60% of affected individuals (Yik et al., 2009; Ebberink et al., 2011). The most common mutant *PEX1* allele is *PEX1-p.Gly843Asp* (*PEX1G843D*), a disease-causing allele found mainly in patients of European origin (Reuber et al., 1997; Collins and Gould, 1999; Walter et al., 2001; Preuss et al., 2002), probably due to a founder effect (Collins and Gould, 1999). The *PEX1G843D* mutation accounts for 12–30% of all PBD-ZSS alleles and contributes to a milder PBD phenotype than patients with *PEX1* truncations, insertions, deletions or other pathogenic variations (Preuss et al., 2002; Crane et al., 2005; Ventura et al., 2016). Moreover, patients carrying the *PEX1G843D* mutant allele or other missense mutant alleles linked to mild PBD-ZSS present less severe biochemical deficiencies and have more functional peroxisomes than do other PBD-ZSS patients due to residual peroxin function in the cells of patients with a mild form of PBD-ZSS (Walter et al., 2001; Preuss et al., 2002; Braverman et al., 2013).

PEX1 is an AAA (ATPase associated with diverse cellular activities)-ATPase that oligomerizes with the PEX6 AAA-ATPase. Together PEX1 and PEX6 form a complex with PEX26 to recycle PEX5, the soluble receptor necessary for peroxisomal protein import from the cytosol into the peroxisome matrix (Ito et al., 2007; Platta and Erdmann, 2007; Smith and Aitchison, 2013). The *PEX1G843D* mutation affects PEX1 folding and attenuates the physical interaction between PEX1 and PEX6 necessary for peroxisomal import by 70% (Geisbrecht et al., 1998; Zhang et al., 2010). Although the variability in disease severity of patients homozygous for the *PEX1G843D* mutation (Poll-The et al., 2004) makes it difficult to predict outcomes solely from genotype, the overall milder phenotype of the homozygous *PEX1G843D* mutation (Berendse et al., 2016) makes it amenable to liver or hepatocyte transplantation (Demaret et al., 2018, 2021) and pharmaceutical therapy to improve quality of life and lifespan for patients carrying this mutation (Zhang et al., 2010).

High-throughput screening (HTS) of libraries of Food and Drug Administration (FDA)-approved compounds can be employed to streamline the process of identifying novel drug candidates for clinical trials. HTS has been successfully used to identify candidate compounds for therapeutic benefit in a number of diseases, including cancer, amyotrophic lateral sclerosis, and diabetes (Sexton et al., 2010; Willoughby et al., 2013; Joardar et al., 2015). A previous HTS of *PEX1G843D* human fibroblasts identified drug candidates that appeared to improve peroxisome function by stabilizing the mutated PEX1 protein (Zhang et al., 2010); however, no drug to date has passed clinical validation. Regardless, previous studies have suggested that *PEX1G843D* patient defects are amenable to intervention at the cellular level and implicate peroxin stabilization and peroxisome proliferation as mechanisms that drugs could target.

Model organisms have been useful in providing *in vivo* models for drug discovery and/or validation of drugs, and the fruit fly *Drosophila melanogaster* has been adopted to develop HTS protocols using flies with human disease-causing mutations (Willoughby et al., 2013). Peroxins and peroxisome functions are conserved between human and flies (Mast et al., 2011; Faust et al., 2012; Baron et al., 2016; Di Cara et al., 2017, 2019), and *Drosophila* is a well established model organism with which to study human diseases (Sanchez-Martinez et al., 2006; Sen and Cox, 2017) and for drug discovery (Willoughby et al., 2013).

In our study, we performed a HTS of an FDA-approved small molecule library with 1,280 pharmacologically active compounds (LOPAC 1280) to screen for molecules that increased peroxisome number in *PEX1G843D* human fibroblasts. We then performed a double-blind validation of hit compounds and various biochemical assays to select for molecules that rescued peroxisome functions in *PEX1G843D* human fibroblasts. We confirmed the efficacy of the candidate compounds on animal survival and lifespan improvement by testing it on a humanized *Drosophila* model carrying the *PEX1G843D* human mutation. Our work demonstrated that *S*-nitrosoglutathione (GSNO), a nitric oxide (NO) donor that is regarded as an intracellular NO reservoir and as a vehicle for NO throughout the cell (Corpas et al., 2013), increases peroxisome numbers in *PEX1G843D* mutant fibroblasts, improves the overall biochemical activities associated with peroxisomes in these mutant fibroblasts, and increases the survival and extends the lifespan of humanized flies with the *PEX1G843D* mutation, thereby opening up the possibility of developing GSNO into a clinical therapy for mild PBD-ZSS patients.

## Materials and Methods

### Cell Culture

Human wild-type (WT) fibroblasts and *PEX1G843D* mutant fibroblasts were kind gifts of Dr. Nancy Braverman (McGill University). Informed consent for research was obtained from the Office of Human Research Administration at the Kennedy Krieger Institute (Baltimore, MD, United States) to the Peroxisome Disease Laboratory, McGill University under Clinical Research Centre grants RR0052 and RR00722. Cells at passages 5–9 were cultured at 37°C in 5% CO_2_ in DMEM with 4.5 g D-Glucose/L, 4.5 g L-Glutamate/L, and 110 mg sodium pyruvate/L supplemented with 10% Fetal Bovine Serum (Hyclone, Thermo Fisher), 50 U penicillin/mL, and 50 μg streptomycin sulfate/mL. PCR amplification using the MycoSensor PCR Assay Kit (Agilent) detected no mycoplasma contamination of cultures.

### Fly Stocks and Husbandry

*Drosophila* strains were cultured at 25°C on standard Bloomington Drosophila Stock Center (BDSC) corn meal medium supplemented with soy powder. For compound treatment experiments, food was supplemented with 50, 100, or 500 μM T6. All compound concentrations tested showed an increase in lifespan of *P*{*UAS- PEX1G843D*} flies compared to the same flies raised on food supplemented only with vehicle (DMSO). The ubiquitous driver line *P*{Ubi-GAL4.U}2/CyO (32551) was purchased from BDSC. *P*{*UAS- PEX1G843D*} mutants were allowed to lay eggs on apple juice agar plates for 2 days. Each day mutant embryos were collected every 2 h and transferred to compound-supplemented medium, and control medium embryos were incubated on apple juice agar plates at 25°C. After 24 h, hatched larvae were transferred to standard corn meal medium or corn meal medium supplemented with compound for oral treatment throughout development. Surviving animals were counted at the same time each day.

### LOPAC 1280 Library Reformatting

Compounds from the LOPAC 1280 library (Sigma-Aldrich) were used at a final concentration of 25 μM in the screen. To minimize pipetting during the screen, we reformatted the original library from a 96-well format into a 384-well format as outlined below:

• Compounds at 10 mM in DMSO were originally in 16 96-well plates (columns 1 and 12 empty). Compounds were reformatted in four 384-well plates (columns 1, 12, 13, and 24 empty).
• 384-well plates were prepared for compound dilution. Each well (except column 12) was filled with 99 μL of serum-free medium and 1 μL of a compound from the 10 mM stock library. 1 μL of DMSO was added to 99 μL of serum-free medium in columns 1, 13, and 24.
• 100 μL of betaine (400 mM stock solution in serum-free medium) was added to the wells in column 12.

### High-Throughput Screening, Immunofluorescence, and Imaging

On day 1, wild-type (WT) and *PEX1G843D* fibroblasts were trypsinized with 2.5% trypsin (Gibco) and seeded onto μClear (Greiner Bio-One) 384-well plates (500 cells per well in 40 μL of medium). On day 2, compounds (13.4 μL of the 1:100 dilution of the stock in DMEM) were added to wells containing *PEX1G843D* fibroblasts, while 13.4 μL of the control drug betaine were added to columns 12 and 13. Four μL of DMSO (vehicle) were added to columns 1, 13, and 24. Untreated WT fibroblasts were used as a positive control for the detection system. Each compound, and positive and negative controls, were tested in duplicate. Cell plates were incubated for 2 days at 37°C and 5% CO_2_. On day 5, cells were fixed in 4% formaldehyde in 1× phosphate buffered saline (PBS) for 30 min, washed once with PBT 0.05% (PBS containing 0.05% Triton X-100) for 2 min, permeabilized in PBT 0.1% (PBS containing 0.1% Triton X-100) for 3 min, washed in PBT 0.05% for 2 min and blocked for 40 min in PBT 0.05% containing 3% BSA. Rabbit antibodies to the C-terminal Peroxisome Targeting Signal Type 1 (PTS1), Ser-Lys-Leu (SKL) (Szilard et al., 1995) were added at 1:200 dilution in 0.3% BSA. Plates were incubated overnight at 4°C. On day 6 primary antibody was removed, and cells were washed in PBT 0.05% three times and incubated in Alexa Fluor 488 anti-rabbit secondary antibody (Abcam, 1:1,000 dilution) and DAPI (1 μg/mL) to detect DNA for 30 min at room temperature. Cells were then washed twice in PBT 0.05% (2 min each wash) and once with PBS. Cells were imaged in PBS. The screen was done at the High Content Analysis Core, University of Alberta, Faculty of Medicine and Dentistry. All pipetting was done using the PerkinElmer Janus Standard Deck automated liquid handling system enclosed in a Class II biosafety cabinet. Plates were imaged using the Operetta High Content Imaging System (PerkinElmer).

A primary screen quantifying the number of SKL-positive puncta per cell area was done using automated Harmony Software (PerkinElmer) as follows:

(1) Find nuclei based on intensity of nuclear staining.
(2) Count total number of nuclei.
(3) Identify the cytoplasm of each cell based on the intensity of Alexa Fluor 488 staining surrounding individual nuclei.
(4) Exclude cells on the edges of each image to reduce the chance that a cell is counted multiple times.
(5) Select a region of the cell in which to quantify spots. The set region was 10 pixels in from the border and including the nucleus.
(6) Calculate the area of the cell region for all cells.
(7) Find spots in the selected cell region. This quantifies the spots (spots/μm^2^). Each spot is detected as a small region on the image having an intensity greater than the intensity of the surrounding region.
(8) Calculate the morphological properties of the spots to get the spot area.
(9) Create a formula to calculate the number of spots per mean cell area.

We applied *z*-score analysis to normalize the number of spots/cell area across the entire screen. *Z*-scores were calculated by subtracting the number of spots/cell area value for each compound by the plate median value and dividing by the standard deviation of the plate. *Z*-scores assume normal distribution and represent the standard deviation of every value from the plate median for each treatment. *Z*-scores with values above 2 represent the 99% confidence interval. *Z*-scores with values above 1.96 represent the 95% confidence interval.

This initial analysis identified 123 compounds with *z*-scores above 2 (Supplementary Table 1). To validate the accuracy of the software, we performed a double-blind screen and confirmed by visual analysis microscopic images of the 11 compounds that produced a greater than 10% increase in the number of SKL-positive puncta per cell area compared to vehicle-treated cells (Supplementary Table 2). Only compounds showing an increase of more than 25% SKL-positive puncta per cell area compared to vehicle-treated cells were chosen for further validation.

For secondary screening, WT fibroblasts and *PEX1G843D* fibroblasts were seeded onto round glass coverslips at a density of 100,000 cells per well in a 24-well plate containing growth medium. The following day, the medium was replaced and supplemented with 25 μM of each compound selected in the primary screen (Supplementary Table 1), 0.4% DMSO (negative control), or 100 mM betaine (positive control). Medium added to WT fibroblasts contained no compound. Cells were incubated with each compound for 2, 3, or 6 days. For prolonged treatments, medium was changed every 2 days. For compound titration studies, cells were treated with 5, 10, 25, 35, or 50 μM of compound for 2 days.

After each treatment, cells were processed for indirect confocal immunofluorescence (IF) microscopy. In brief, fibroblasts were fixed for 30 min in 4% paraformaldehyde in PBS, rinsed twice in PBST (PBS, 0.1% Triton X-100), and blocked for 1 h in 5% normal goat serum (Sigma-Aldrich) before incubation for 16 h at 4°C with primary antibody. After four washes in PBST, cells were incubated with secondary antibody for 16 h at 4°C, washed four times in PBST, and mounted in Prolong-Gold (Thermo Fisher). Images were captured using a C9100 camera (Hamamatsu) at 130-μm vertical spacing using a 100× oil immersion objective (NA = 1.4) on a Zeiss AxioObserver M1 microscope coupled to an ERS spinning disk confocal microscope (PerkinElmer). Primary antibodies were mouse anti-ABCD3 (Sigma-Aldrich) to stain peroxisomal membranes (Imanaka et al., 2000), rabbit anti-SKL to stain the peroxisomal matrix (Szilard et al., 1995) and mouse anti-KDEL (Santa Cruz Biotechnology) to stain the ER. Secondary antibodies were Alexa Fluor 568 donkey anti-mouse and Alexa Fluor 488 donkey anti-rabbit (Abcam).

Confocal image stacks were processed to remove noise and reassign blur using a classical maximum likelihood estimation confocal algorithm provided by Huygens Professional Software and an experimentally determined point spread function constructed from multiple images of 0.1 μm Tetraspeck beads (Life Technologies). Individual peroxisome volume and average number of peroxisomes per cell were calculated using the Spots function of Imaris 8 software (Bitplane). Each calculation (volume and size) represents a mean of 20 independent cells from each biological sample. Each experiment had at least three biological replicates. For the final validation of the GSNO hit we processed and analyzed images for over 600 biological replicates with Imaris 8 software or with the automated Harmony software. The number was established by G^∗^Power analysis (Faul et al., 2007), a statistical software that indicates the number of biological replicates required to claim robust statistical significance based on the standard deviation in a small sample of the analyzed population.

ER expansion was determined by measuring the corrected total anti-KDEL IF and comparing the fluorescence intensity of KDEL-positive regions between cells of the reported genotype and under the reported condition. Analysis of IF images was done as follows:

(1) Outline desired cell with Freehand ROI tool.
(2) Set desired parameters by going to Analyze > Set Measurements > Select: Area, Integrated Density, and Mean Gray Value.
(3) Go to Analyze > Measure.
(4) Copy data into a spreadsheet.
(5) Then select a small area of the image that has no fluorescence (background).
(6) Analyze > Measure for that region. Copy data into spreadsheet.
(7) Repeat steps 3–6 for 20 cells and background regions.
(8) Calculate the mean fluorescence of background readings.
(9) Calculate corrected total cell fluorescence (CTCF) = Integrated Density − (Area of Selected Cell × Mean Fluorescence of Background Readings).
(10) Calculate CTCF for each cell.

### MitoTracker Red and LysoTracker Red Staining

Wild-type and *PEX1G843D* fibroblasts were seeded at a density of 100,000 cells per well onto round glass coverslips in a 24-well plate containing medium. The following day, the medium was replaced with medium containing 25 μM of each compound selected in the primary screen (Supplementary Table 2) or 0.4% DMSO (vehicle). Medium without compound was added to WT fibroblasts. On day 2, MitoTracker Red or LysoTracker Red was added to wells at a final concentration of 25 nM or 25 μM, respectively, for 30 min at 37°C. The medium was then removed, and cells were fixed for 30 min in 4% paraformaldehyde in PBS, rinsed twice in PBS and mounted in Prolong-Gold (Thermo Fisher). Images were captured using a C9100 camera at 130-μm vertical spacing using a 100× oil immersion objective (NA = 1.4) on a Zeiss AxioObserver M1 microscope coupled to an ERS spinning disk confocal microscope. Determination of mitochondria shape and quantification of mitochondria networks per cell genotype and under each condition were performed using ImageJ and the macro MiNA-master following a published protocol (Valente et al., 2017). The number of LysoTracker Red-positive puncta per cell genotype and under each condition was determined as described (Di Cara et al., 2018).

### Cloning

The coding sequence for the mutant human protein PEX1G843D in the plasmid pcDNA3-PEX1G843D (a gift from Dr. Nancy Braverman, McGill University) was amplified using the primers *PEX1*-Forward 5′-CACCATGTGGGGCAGCGAT and *PEX1*-Reverse 5′-TTATGCTAAAGTTACTTTCT and Phusion High Fidelity DNA polymerase (Thermo Fisher). PCR product was directionally cloned into the pENTR/D entry vector by TOPO cloning (Invitrogen) for the Gateway System (Life Technologies). Plasmid inserts were verified by sequencing. Inserts were then recombined into pTW destination vectors (Drosophila Gateway Vector Collection) using LR clonase II (Life Technologies). The purified final recombinant DNA construct was provided to BestGene^1^ for injection into the *Drosophila* strain *y^1^ w^67c23^*; *P{CaryP}attP40* strain to establish a transgenic fly line able to express PEX1G843D using the Gal4/UAS system (Brand and Perrimon, 1993).

### Lipid Analysis

One million cells of each genotype and under each condition were resuspended in 1 mL of PBS buffer and sonicated for 5 min using a BioRuptor (Diagenode) at low power to produce a cell lysate. The protein content of cell lysates was determined using a Qubit II fluorimeter (Thermo Fisher). One million cells yielded 1 mg total protein. Lipids were extracted using chloroform:methanol (2:1) as described (Folch et al., 1957). Extracts were centrifuged at 3,400 × *g*, and the chloroform phase containing lipids was passed through a sodium sulfate column (GE Healthcare). Five μg of heptadecane (C_17_) in chloroform was used as an internal control. The eluate was dried under nitrogen gas and resuspended in 100 μL of HPLC-grade hexane. Ten μL of material were injected into an Agilent 6890 gas chromatograph with a flame-ionization detector.

### Assay for Peroxisomal β-Oxidation Activity

Fibroblasts were homogenized in 0.25 M STKM buffer (0.25 M sucrose, 25 mM HEPES-KOH, pH 7.4, 25 mM KOAc, 5 mM MgCl_2_, 0.1 mM EDTA, 1× Roche Complete Protease Inhibitor, 1 mM DTT) and sonicated for 5 min using a BioRuptor (Diagenode) at low power. Lysates were centrifuged at 1,000 × *g* for 10 min at 4°C, and the resultant supernatant was centrifuged at 20,000 × *g* for 20 min at 4°C to yield a pellet enriched for peroxisomes. The protein content of peroxisome-enriched subcellular fractions was determined using a Qubit II fluorimeter. Peroxisomal ß-oxidation enzymatic activity of subcellular fractions was measured essentially as described (Lazarow and de Duve, 1976).

### Assay for Catalase Activity

Fibroblasts were homogenized in 0.25 M STKM buffer and sonicated for 5 min using a BioRuptor at low power. Lysates were centrifuged at 1,000 × *g* for 10 min at 4°C, and the resultant supernatant was centrifuged at 20,000 × *g* for 20 min at 4°C to yield a pellet enriched for peroxisomes. Catalase activity of subcellular fractions was determined using the Amplex Red Catalase Assay Kit (Thermo Fisher) and normalized to protein amount. Experiments were done in triplicate.

### Protein Analysis and Immunoblotting

Fifty μL of cold Ephrussi-Beadle Ringer’s solution supplemented with 10 mM EDTA, 10 mM DTT, 1× complete protease inhibitor, and 1× PhosStop phosphatase inhibitor (Roche) were added to 3 × 10^6^ pelleted cells, and a cell lysate was prepared by sonication for 5 min using a BioRuptor at low power. Protein amounts of lysates were determined using a Qubit II fluorimeter. Twenty-five μL of 3× SDS-PAGE Buffer (Bio-Rad) containing 10 mM DTT at 70°C were added to the lysate and placed in boiling water for 10 min. Samples were resolved by SDS-PAGE on 10% acrylamide gels and transferred to nitrocellulose membranes (Bio-Rad). Membranes were blocked with 5% skimmed milk powder in TBS + Tween 20 (TBSTw) (150 mM NaCl, 20 mM Tris-HCl, pH 7.5, 0.05% Tween 20) for 1 h and incubated for 16 h with primary antibody in TBSTw. After washing three times for 5 min each with TBSTw, membranes were incubated with HRP-conjugated secondary antibody (Bio-Rad) at 1:10,000 dilution for 1 h at 24°C. Membranes were washed as above, and HRP activity was detected by enhanced chemiluminescence (Amersham). Primary antibodies were rabbit anti-3-ketoacyl-CoA thiolase (Bodnar and Rachubinski, 1990) and mouse anti-α-tubulin (Sigma-Aldrich).

## Results

### Identification of Candidate Compound T6 by High-Throughput Screening of the LOPAC 1280 Library

To identify compounds that improve peroxisome function in patients carrying the *PEX1G843D* mutation, we performed a HTS of the 1280-compound library of pharmacologically active compounds (LOPAC 1280) on *PEX1G843D* mutant human fibroblasts. To do the HTS, *PEX1G843D* fibroblasts and WT fibroblasts were treated either with 25 μM of each compound from the library or with 0.4% vehicle (DMSO). As a positive control, fibroblasts were treated with 100 mM betaine, a compound previously reported to increase peroxisome number in *PEX1G843D* fibroblasts (MacLean et al., 2019). After 3 days, we performed indirect immunofluorescence (IF) microscopy using an antibody against the C-terminal Peroxisome Targeting Sequence Type 1 Ser-Lys-Leu (SKL), the canonical marker for peroxisomal matrix proteins (Szilard et al., 1995). We imaged the fibroblasts using a high-content analysis imager with automated cell counting software to identify compounds that increased the number of SKL-positive puncta in compound-treated *PEX1G843D* fibroblasts compared to untreated *PEX1G843D* fibroblasts.

**FIGURE 1:**
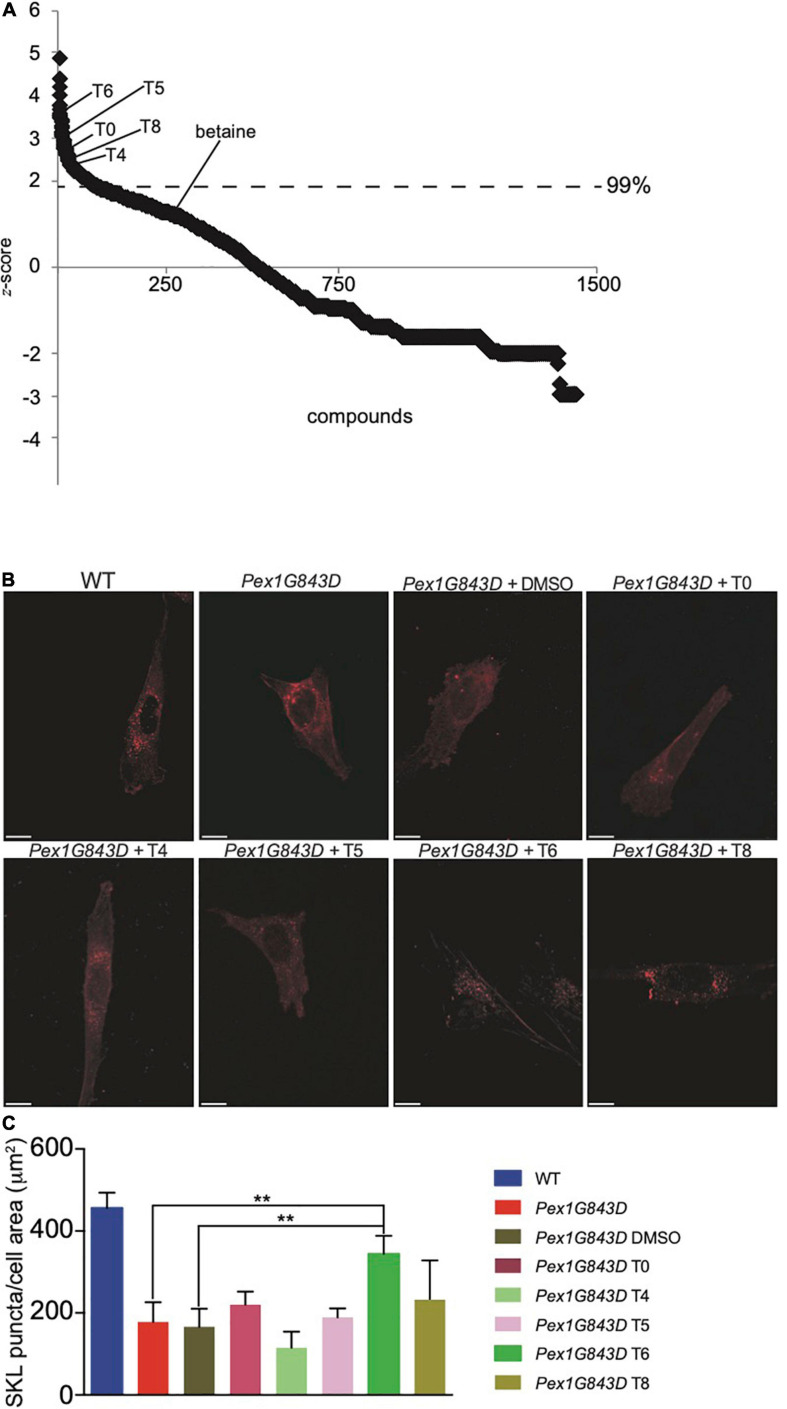
HTS of the LOPAC 1280 library identified candidate compounds to rescue peroxisome number in *PEX1G843D* human fibroblasts. **(A)** Statistical analysis of candidate compounds measured by *z*-score. Significant activity threshold was *z* > 2. Note that betaine, a compound known to increase peroxisome numbers and used as a positive control, was below threshold in this screen. **(B)** Detection of SKL-positive puncta by indirect IF microscopy in fibroblasts of the reported genotypes and under the designated conditions. Images of *PEX1G843D* fibroblasts treated with some candidate compounds are shown and compared to the images of WT fibroblasts, untreated *PEX1G843D* fibroblasts, and *PEX1G843D* fibroblasts treated with vehicle (0.04% DMSO). Note that treatment with DMSO alone does not result in increased numbers of SKL-positive puncta. Scale bars, 10 μm. **(C)** Histogram reporting the quantification of SKL-positive puncta per cell area (μm^2^) for cells of each genotype and under each condition observed by indirect IF microscopy. Error bars represent standard deviation. Statistical significance was calculated using 2-way ANOVA. **p < 0.01.

We quantified the number of SKL-positive puncta per cell area for each experimental and control well using Harmony image analysis software. We calculated the *z*-score of the resulting quantification of SKL-positive puncta for all compounds tested to establish thresholds of significance and to enable plate-to-plate comparisons. The *z*-score analysis separated chemical compounds by efficacy as a deviation from baseline (Figure 1A). The threshold *z*-score of 2 was dictated by the 99% confidence interval of the *z*-score. The positive control compound betaine was below the threshold *z*-score of 2 (Figure 1A), indicating that all hit compounds identified in this screen pipeline were more efficacious than betaine. Each compound hit from the primary HTS was visually inspected for increased numbers of peroxisome puncta in a double-blind analysis. Of the 123 compounds identified (Supplementary Table 1), 11 compounds were validated by secondary analysis, and structural and functional information was compiled for each of the compounds, which targeted a wide-range of cellular functions (Supplementary Table 2). We assigned arbitrary identifiers, T0-T10, to each compound and performed an indirect IF microscopy screen to confirm an increase in SKL-positive puncta with compound treatment (Figures 1B,C). We tested every compound that showed greater than 25% rescue of the number of SKL-positive puncta in our secondary screen. Indirect IF microscopy analyses revealed that only treatment with T6, the nitric oxide donor GSNO (Stolz et al., 2002; Corpas et al., 2013, 2017, 2021), reproducibly increased the number of SKL-positive puncta in *PEX1G843D* fibroblasts almost to numbers observed in WT fibroblasts (Figures 1B,C and Supplementary Figure 1A). We therefore further characterized the effect of T6 treatment on peroxisome numbers and functions in *PEX1-G843D* fibroblasts.

### T6 Treatment of *PEX1G843D* Fibroblasts Leads to Increased Peroxisomal Matrix Protein Import and Increased Peroxisome Biogenesis

**FIGURE 2:**
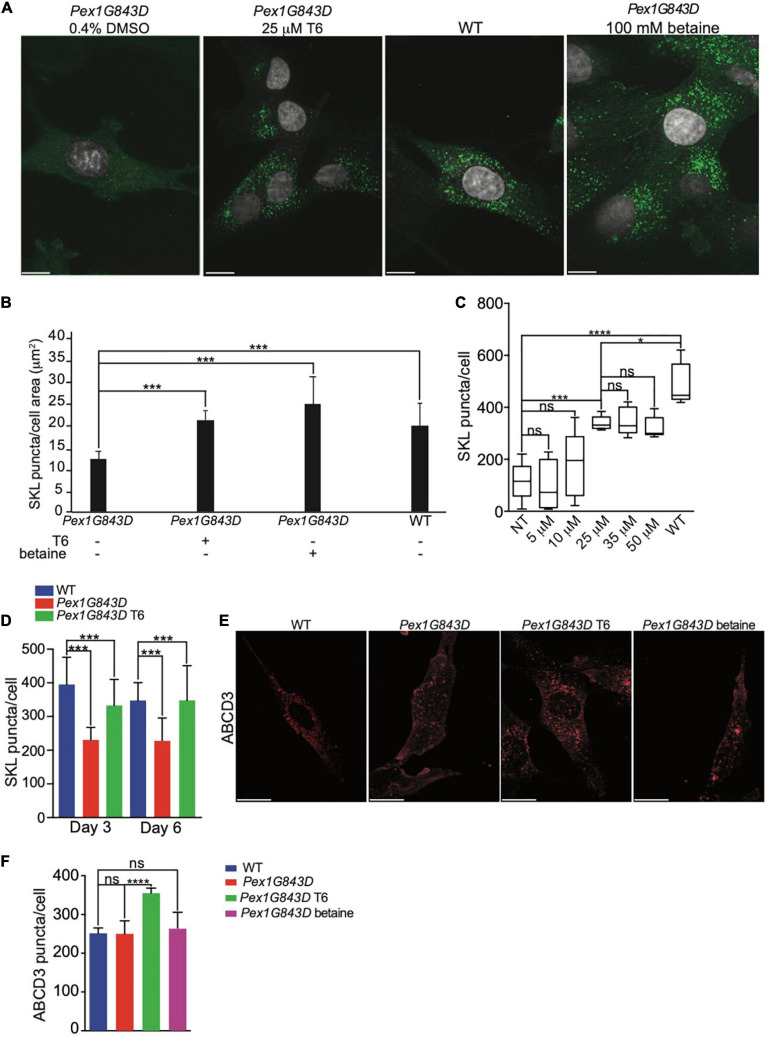
Treatment with compound T6 enhances peroxisomal matrix protein import and increases peroxisome number in *PEX1G843D* fibroblasts. **(A)** Indirect IF microscopy of *PEX1G843D* fibroblasts with anti-SKL antibodies to compare the effects of treatment with T6 to treatment with betaine (positive control) or vehicle (DMSO). Green puncta, anti-SKL. Gray, nuclei. Scale bars, 10 μm. **(B)** Histogram reporting the quantification of SKL-positive puncta per unit of cell area (μm^2^) for each genotype and condition. Error bars represent standard deviation (*N* = 646). Statistical significance was calculated using 2-way ANOVA. ***p < 0.001. **(C)** Quantification of the number of SKL-positive puncta in *PEX1G843D* fibroblasts in response to increasing concentrations of T6. Error bars report the standard deviation (*N* = 25). Statistical significance was calculated using 1-way ANOVA. ***p < 0.001. ns, not significant. NT, not treated. **(D)** T6 treatment beyond 3 days does not lead to correspondingly increased numbers of peroxisomes in *PEX1G843D* fibroblasts. Histogram reports the quantification of SKL-positive puncta per cell for each genotype and condition. Error bars represent standard deviation (*N* = 25). Statistical significance was calculated using 2-way ANOVA. ***p < 0.001. **(E)** Indirect IF microscopy of *PEX1G843D* fibroblasts with anti-ABCD3 antibodies comparing treatment with T6 to treatment with betaine. Scale bars, 10 μm. **(F)** Histogram reporting the quantification of ABCD3-positive puncta in cells of each genotype and condition as in **(E)**. Note that treatment with T6 led to increased numbers of ABCD3-positive puncta in *PEX1G843D* fibroblasts relative to numbers in WT cells. Error bars represent standard deviation (*N* = 25). Statistical significance was calculated using 2-way ANOVA. *p < 0.05, ***p < 0.001, ****p < 0.0001. ns, not significant.

We investigated how T6 might increase peroxisomal SKL-positive puncta and therefore increase peroxisomal protein import in *PEX1G843D* fibroblasts. As the *PEX1G843D* mutation only partially impairs peroxisomal protein import, we hypothesized that T6 can aid peroxisomal import of proteins containing a PTS1 either by improving peroxisomal protein import *per se* or by increasing the *de novo* biogenesis of peroxisomes and, as a consequence, the overall activity of peroxisomes in the mutant cells. To confirm that T6 treatment impacts peroxisomal protein import, we performed indirect IF microscopy of peroxisomal puncta labeled by anti-SKL antibodies in *PEX1G843D* fibroblasts treated with 25 μM T6, vehicle (0.4% DMSO, negative control), 100 mM betaine (positive control), together with WT fibroblasts that function as reference. To obtain the highest statistical significance on the efficacy of T6, we performed an *a priori* sample size analysis, using G^∗^Power analysis, a statistical software that indicates the number of biological replicates necessary to obtain robust statistical significance based on the standard deviation of the analyzed population (Faul et al., 2007) (Figure 2A). Imaris quantification of the number of SKL-positive puncta per cell area showed a statistically significant increase in the number of SKL-positive puncta in T6-treated *PEX1G843D* fibroblasts, similar to the numbers of SKL-positive puncta in WT cells in 646 independent biological replicates (Figure 2B), strongly supporting the conclusion that peroxisomal protein import was improved in *PEX1G843D* fibroblasts upon T6 treatment. The number of SKL-positive puncta in T6-treated *PEX1G843D* fibroblasts increased significantly with respect to the number of puncta in untreated *PEX1G843D* fibroblasts at concentrations of T6 25 μM and higher, although concentrations of T6 greater than 25 μM did not lead to correspondingly increased numbers of SKL-positive puncta in *PEX1G843D* fibroblasts treated with 25 μM T6 (Figure 2C). Accordingly, we used T6 at 25 μM concentration for all subsequent analyses. To test whether the observed effect on peroxisomal protein import improved with longer treatment of *PEX1G843D* fibroblasts with T6, we increased the period of T6 treatment from 3 to 6 days and measured the number of SKL-positive puncta in treated *PEX1G843D* fibroblasts compared to untreated *PEX1G843D* fibroblasts and to WT fibroblasts (Figure 2D). Although the number of SKL-positive puncta in T6-treated *PEX1G843D* fibroblasts increased in number relative to untreated *PEX1G843D* fibroblasts at both 3 and 6 days, increasing the time of treatment with T6 from 3 to 6 days did not produce a statistically significant increase in the number of SKL-positive puncta in *PEX1G843D* fibroblasts compared to WT fibroblasts. Therefore, the effects of T6 treatment did not appear to depend on the length of time of treatment beyond 3 days.

Indirect IF microscopy of cells labeled with antibodies to ATP Binding Cassette Subfamily D Member 3 (ABCD3), a peroxisomal membrane protein, showed that T6-treated *PEX1G843D* fibroblasts exhibited more ABCD3-positive puncta than untreated *PEX1G843D* fibroblasts, and the numbers of ABCD3-positive puncta in T6-treated *PEX1G843D* fibroblasts were similar to the numbers of ABCD3-positive puncta in WT fibroblasts (Figures 2E,F), indicating that T6 treatment increases the number of peroxisome structures by increased peroxisome biogenesis or enhanced peroxisome stability. Interestingly, treatment of *PEX1G843D* fibroblasts with betaine did not increase the number of ABCD3-positive puncta (Figures 2E,F). However, T6 treatment did increase the number of ABCD3-positive structures also in WT cells (Supplementary Figure 1A), suggesting that T6 increases the number of peroxisomes in *PEX1G843D* fibroblasts at least in part by enhanced peroxisome biogenesis or reduced peroxisome degradation.

### T6 Treatment Leads to Increased Peroxisomal Biochemical Activities in *PEX1G843D* Fibroblasts

**FIGURE 3:**
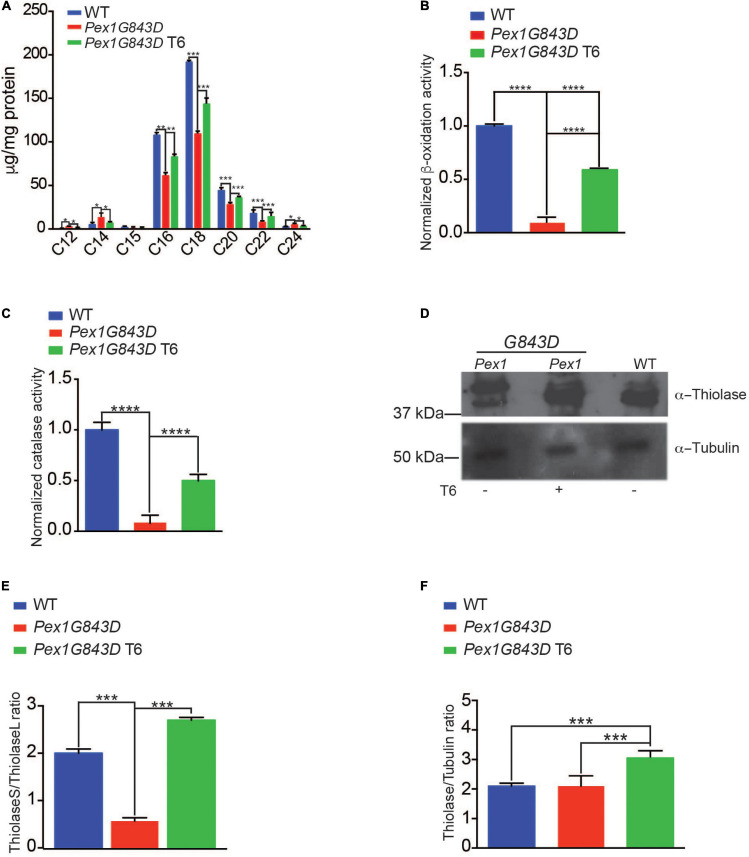
T6 treatment improves peroxisomal function of *PEX1G843D* fibroblasts. **(A)** Fatty acid (FA) profiles in cells of the reported genotypes and under the reported conditions. FA amounts were normalized to mg protein for each sample (*N* = 4). Error bars represent standard deviation. Statistical significance was calculated using 2-way ANOVA. ***p < 0.001, **p < 0.01, *p < 0.05. **(B)** Treatment of *PEX1G843D* fibroblasts with T6 leads to increased peroxisomal β-oxidation activity in a peroxisome-enriched subcellular fraction compared to the same fraction from untreated *PEX1G843D* fibroblasts. Error bars represent standard deviation (*N* = 4). Statistical significance was calculated using 2-way ANOVA. ***p < 0.001. **(C)** Treatment of *PEX1G843D* fibroblasts with T6 leads to increased catalase activity in a peroxisome-enriched subcellular fraction compared to the same fractions from untreated *PEX1G843D* fibroblasts. Error bars represent standard deviation (*N* = 4). Statistical significance was calculated using 2-way ANOVA. ***p < 0.001. **(D)** Representative immunoblot showing increased amounts of mature imported ThiolaseS (42 kDa) in T6-treated *PEX1G843D* fibroblasts versus untreated *PEX1G843D* fibroblasts. Tubulin served as a control for protein loading. Immunoblotting was repeated three times. Equal amounts of protein (500 μg) were loaded in each lane. **(E)** Histogram reporting quantification of immunoblot results of the ratio ThiolaseS/ThiolaseL for each genotype and under each condition. Error bars represent standard deviation (*N* = 3). Statistical significance was calculated using 2-way ANOVA. ***p < 0.001. **(F)** Histogram reporting increased levels of thiolase in T6-treated *PEX1G843D* fibroblasts versus untreated *PEX1G843D* fibroblasts or WT fibroblasts. Error bars represent standard deviation (*N* = 3). Statistical significance was calculated using 2-way ANOVA. ***p < 0.001.

As treatment of *PEX1G843D* fibroblasts with T6 led to increased numbers of peroxisome structures as seen by indirect IF microscopy, we performed a series of biochemical analyses to investigate if treatment with T6 also led to increased peroxisomal function and also increased peroxisomal protein import. One key metabolic function of peroxisomes is the metabolism of very long-chain fatty acids (VLCFAs) (Wanders and Waterham, 2006), which are fatty acids (FAs) equal to or greater than C_22_ in length. As such, improved peroxisomal FA metabolism can be monitored by investigating the FA profiles of cells under various conditions. We performed gas chromatography to analyze and compare the FA profiles of T6-treated *PEX1G843D* fibroblasts, untreated *PEX1G843D* fibroblasts, and WT fibroblasts (Figure 3A). FA quantification in cells of each genotype and under each condition was normalized to total protein. Untreated *PEX1G843D* mutant fibroblasts had decreased medium-chain fatty acids (MCFAs, C_16__–__22_), decreased C_22_ FAs and slightly increased C_24_ FAs relative to WT fibroblasts (Figure 3A). T6 treatment increased C_22_ FAs and decreased C_24_ FAs in *PEX1G843D* fibroblasts compared to the levels in WT fibroblasts and also rescued the levels of MCFAs to the levels found in WT fibroblasts (Figure 3A), altogether indicating improved FA metabolism in T6-treated *PEX1G843D* fibroblasts. T6 treatment of *PEX1G843D* fibroblasts also improves peroxisomal ß-oxidation activity over untreated fibroblasts (Figure 3B). leads not only to increased numbers of peroxisomes but also rescues peroxisomal ß-oxidation activity levels in these fibroblasts to levels approaching those observed in WT fibroblasts.

To obtain further support for the overall improvement in peroxisomal function in T6-treated versus untreated *PEX1G843D* fibroblasts, we monitored the relative activities of peroxisomal ß-oxidation (Lazarow and de Duve, 1976) and of peroxisomal catalase, the enzyme responsible for the breakdown of hydrogen peroxide into molecular oxygen and water (Wanders and Waterham, 2006). Treatment of *PEX1G843D* fibroblasts with T6 led to significantly increased peroxisomal ß-oxidation activity (Figure 3B) and catalase activity (Figure 3C) in a subcellular fraction enriched for peroxisomes compared to the fraction from untreated *PEX1G843D* fibroblasts, providing additional indicators of the recovery of peroxisome function in T6-treated *PEX1G843D* fibroblasts.

To provide additional support for the rescue of peroxisome activity by treatment of *PEX1G843D* fibroblasts with T6, we performed immunoblot analysis to monitor thiolase import into the peroxisomal matrix. Peroxisomal thiolase is an enzyme involved in FA ß-oxidation and catalyzes the reversible thiolytic cleavage of 3-ketoacyl-CoA into acyl-CoA and acetyl-CoA. Thiolase is synthesized as a larger precursor in the cytosol and is cleaved to a shorter active form upon import into peroxisomes (Wanders and Waterham, 2006). Immunoblot assays performed on WT fibroblasts, untreated *PEX1G843D* fibroblasts, and T6-treated *PEX1G843D* fibroblasts showed that immature thiolase (44 kDa, ThiolaseL) was the major form detected in untreated *PEX1G843D* fibroblasts (Figure 3D), whereas the amount of mature thiolase (42 kDa, ThiolaseS) increased upon T6 treatment of *PEX1G843D* fibroblasts to levels similar to those observed in WT fibroblasts (Figure 3D). To quantify thiolase maturation, we calculated the ratio of ThiolaseS to ThiolaseL measured from immunoblot signals (Figure 3E). Treatment with T6 significantly increased the ThiolaseS/ThiolaseL ratio (Figure 3E) and overall thiolase amounts (Figure 3F) of *PEX1G843D* fibroblasts, consistent with the improvement in peroxisomal ß-oxidation activity of T6-treated *PEX1G843D* fibroblasts (Figure 3B). Altogether, our findings indicate that T6 treatment improves peroxisomal function in *PEX1G843D* fibroblasts.

### T6 Treatment of *PEX1G843D* Fibroblasts Restores the Size and Structure of the Mitochondrial Compartment Found in WT Fibroblasts

**FIGURE 4:**
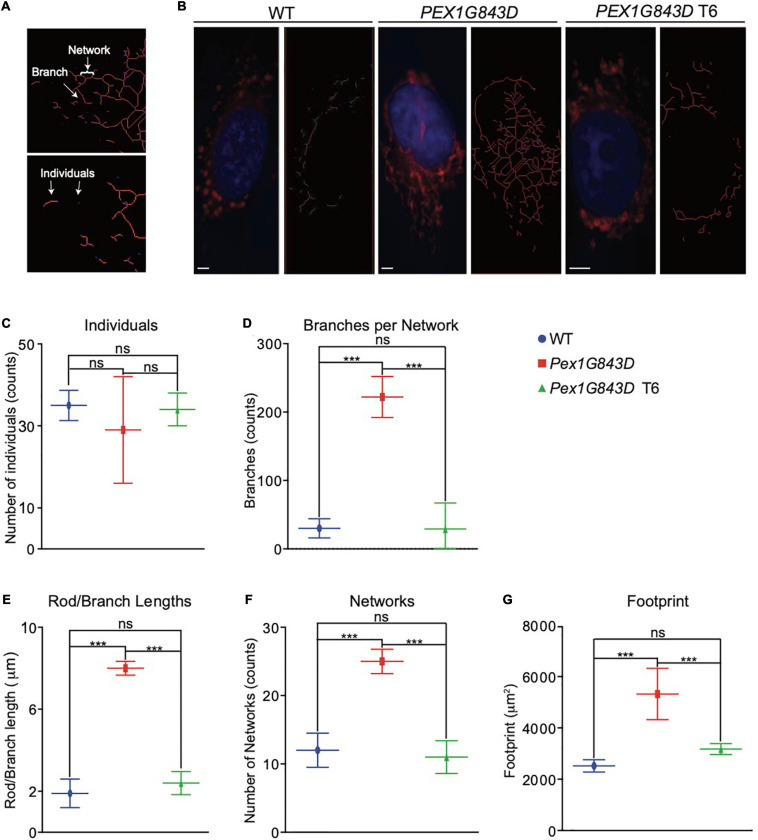
T6 treatment restores the mitochondrial compartment in *PEX1G843D* fibroblasts. **(A)** Mitochondria were stained by MitoTracker Red and observed by fluorescence microscopy. Examples of the two types of mitochondrial features, individuals and networks, recognized by MiNA are shown. Networks can have different numbers of branches. **(B)** Fluorescence images of MitoTracker Red-stained WT fibroblasts, *PEX1G843D* mutant fibroblasts, and T6-treated *PEX1G843D* fibroblasts. The image at right in each pair of images shows skeletonized mitochondria required for analysis by MiNA. DNA was stained with DAPI. Red, mitochondria. Blue, nuclei. Scale bars, 10 μm. Histograms reporting the number of individuals **(C)**, mean branches per network **(D)**, mean rod to branch length **(E)**, number of networks **(F)**, and footprint area per cell **(G)** obtained by MiNA for WT fibroblasts, *PEX1G843D* mutant fibroblasts, and T6-treated *PEX1G843D* fibroblasts. Error bars represent standard deviation (*N* = 25). Statistical significance was calculated using 1-way ANOVA. ***p < 0.001. ns, not significant.

The absence of functional peroxisomes in PBD-ZSS leads to the proliferation of mitochondria and alteration of their structure. These changed mitochondria produce increased amounts of reactive oxygen species (ROS). Increased oxidative stress is posited to contribute significantly to the pathogenesis of PBD-ZSS (Baumgart et al., 2001). We therefore investigated whether there were any changes in mitochondrial morphology in *PEX1G843D* mutant fibroblasts compared to WT fibroblasts and whether these changes could be reversed by treatment of the mutant fibroblasts with T6. We observed mitochondria stained with MitoTracker Red by fluorescence microscopy and used the MiNA (Mitochondrial Network Analysis) platform (Valente et al., 2017) to compare the organization of mitochondria in *PEX1G843D* mutant fibroblasts to that of mitochondria in WT fibroblasts and T6-treated *PEX1G843D* fibroblasts. Mitochondria that were punctate or rod-shaped in appearance were defined as ‘Individuals,’ while mitochondria that were rods with three or more side branches were defined as a ‘Network’ (Figure 4A). MiNA showed that the number of individual mitochondria did not change between WT fibroblasts, *PEX1G843D* mutant fibroblasts, and T6-treated *PEX1G843D* fibroblasts (Figures 4B,C). Mitochondrial networks showed increased complexity in *PEX1G843D* mutant fibroblasts compared to WT fibroblasts, but treatment of *PEX1G843D* fibroblasts with T6 reduced the complexity of their mitochondrial networks to the level of complexity observed in WT fibroblasts (Figures 4B,D–F). The mitochondrial footprint was larger in untreated *PEX1G843D* mutant fibroblasts compared to WT fibroblasts (Figure 4G), suggesting that mitochondrial metabolism is altered in *PEX1G843D* fibroblasts, as expansion of the mitochondrial compartment has been linked to changes in mitochondrial metabolism (McCarron et al., 2013). Again, treatment of *PEX1G843D* fibroblasts with T6 restored the mitochondrial footprint of the mutant fibroblasts to essentially the area of the mitochondrial footprint in WT fibroblasts (Figure 4G). Because NO donors like GSNO (T6) have been associated with remodeling of the endoplasmic reticulum (ER), we also investigated whether there were any differences in ER morphology between WT fibroblasts, *PEX1G843D* mutant fibroblasts, and T6-treated *PEX1G843D* fibroblasts. Indirect IF microscopy using anti-KDEL antibodies that label the ER did not show a change in the size of the ER compartment between cells of the different genotypes and under the different conditions (Supplementary Figures 1D,E). Together, our experiments show that T6 treatment of *PEX1G843D* fibroblasts restores peroxisome functions and the structure and extent of the mitochondrial compartment to at or near what is observed in WT fibroblasts.

### T6 Treatment Reduces the Amount of Autophagy in *PEX1G843D* Fibroblasts

**FIGURE 5:**
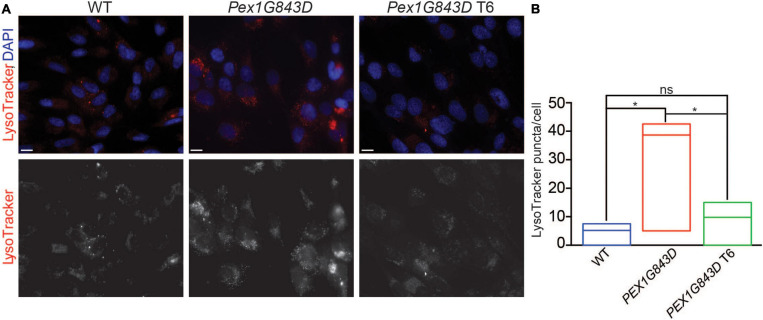
T6 treatment reduces the number of lysosomes in *PEX1G843D* mutant fibroblasts. **(A)** WT fibroblasts, *PEX1G843D* mutant fibroblasts, and T6-treated *PEX1G843D* fibroblasts were stained with LysoTracker Red and observed by fluorescence microscopy. Nuclei were stained with DAPI (blue). Scale bars, 10 μm. **(B)** Histogram reporting the number of LysoTracker Red-stained puncta per cell of each genotype and under each condition. Error bars represent standard deviation (*N* = 20). Statistical significance was calculated using 1-way ANOVA. *p < 0.05. ns, not significant.

Increased pexophagy (peroxisome-specific autophagy) has been suggested as an alternative to defective peroxisome biogenesis as a cause of PBD-ZSS (Law et al., 2017). Loss of the PEX1/PEX6 AAA-complex results in increased ubiquitination of PEX5 on peroxisome membranes, which can then signal pexophagy (Law et al., 2017). Given the potential link between increased pexophagy, PEX1 and PBD-ZSS, we monitored the number of lysosomes in WT fibroblasts, *PEX1G843D* mutant fibroblasts, and T6-treated *PEX1G843D* fibroblasts by fluorescence microscopy of cells stained with LysoTracker Red. *PEX1G843D* mutant fibroblasts have more stained puncta in their cytoplasm compared to WT fibroblasts (Figures 5A,B). T6 treatment of *PEX1G843D* fibroblasts reduced the number of stained puncta to essentially WT numbers (Figures 5A,B). Our data suggest that T6 treatment might increase the number of peroxisomes in *PEX1G843D* mutant fibroblasts by reducing peroxisome damage and/or degradation by pexophagy.

### T6 Treatment Contributes to Increased Survival and Longer Lifespan in a *PEX1G843D* Humanized *Drosophila* Model of PBD-ZSS

**FIGURE 6:**
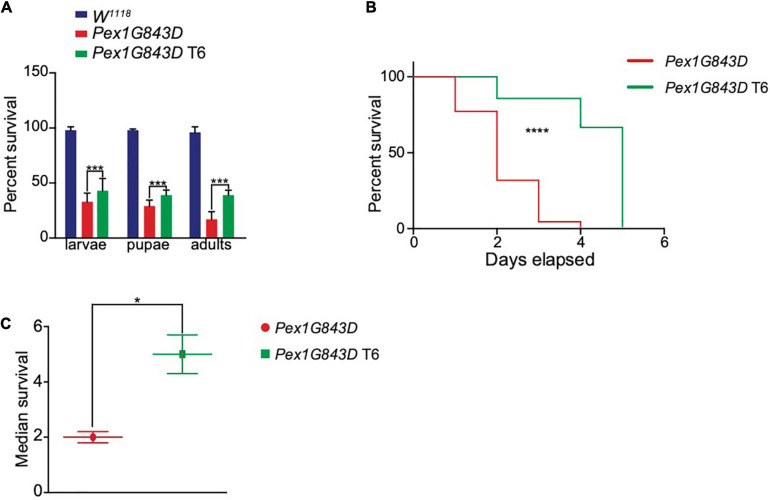
T6 treatment improves survival and extends lifespan in a *Drosophila PEX1G843D* PBD-ZSS model. **(A)** Percent survival of wild-type (*W*^118^), *PEX1G843D* humanized mutant, and T6-treated *PEX1G843D* humanized mutant *Drosophila* at each stage of fly development. 200 eggs per genotype and condition were collected at the start. Note that T6 significantly increased the survival of *PEX1G843D* humanized mutant flies at each stage of development. Statistical significance was calculated using 1-way ANOVA. ***p < 0.001. **(B)** Survival curves of age-matched untreated and T6-treated *PEX1G843D* humanized *Drosophila* mutant. Statistical significance was calculated using the Log-rank test and the Gehan–Breslow–Wilcoxon test. ****p < 0.0001. **(C)** Median survival of age-matched untreated flies and T6-treated *PEX1G843D* humanized *Drosophila* mutant flies. Statistical significance was calculated using 1-way ANOVA. *p < 0.05.

The *PEX1G843D* mutation only partially impairs PEX1 function, a contributing factor in the relatively mild severity of the PBD-ZSS phenotype observed in patients with this mutation. For this reason, there is significant potential for pharmaceutical intervention to improve the lifespan of patients carrying the *PEX1G843D* mutation. To investigate whether T6 could improve developmental survival and lifespan *in vivo*, we generated a humanized *Drosophila* PBD-ZSS disease model carrying the human *PEX1G843D* mutation. To follow the effects of T6 administration on *PEX1G843D* mutant fly development and survival, we collected *PEX1G843D*-mutant eggs and WT eggs and allowed them to develop on normal corn meal medium or T6-supplemented corn meal medium. The percent survival of larvae, pupae, and adult *PEX1G843D* flies significantly increased with T6 supplementation (Figures 6A,C), although not to WT levels, indicating T6 treatment improved survival at each stage of the fly life cycle. Mutant flies raised on T6-supplemented corn meal medium showed a modest but significant improvement in lifespan compared to mutant flies raised on unsupplemented corn meal medium with an increase in median survival of 3 days (Figures 6B,C). Therefore, T6 treatment caused significant but modest improvements on the survival and lifespan of flies with the humanized *PEX1G843D* mutation.

## Discussion

Our study aimed to identify FDA-approved compounds that improve peroxisomal function of *PEX1G843D* PBD-ZSS mutant fibroblasts via HTS. Our HTS led to the identification of the compound GSNO, a NO donor, as a candidate therapeutic for the restoration of peroxisome number and function affected by the *PEX1G843D* mutation.

The HTS reported here initially identified 123 compounds out of the 1280-compound LOPAC library that increased the number of SKL-positive puncta in *PEX1G843D* fibroblasts above the *z*-score threshold specified by the 99% confidence interval. However, we were only able to confirm 11 compounds that increased the number of peroxisome puncta upon visual inspection, and only one compound that could reproduce this result upon repeated IF microscopy experiments using antibodies to peroxisomal matrix proteins (SKL) and to the peroxisomal membrane protein, ATP Binding Cassette Subfamily D Member 3 (ABCD3). The number of false positive hits is not unexpected, as *PEX1G843D* fibroblasts have been reported to exhibit a high degree of mosaicism in regards to their numbers of peroxisomes (Preuss et al., 2002; Zhang et al., 2010). Therefore, the identification of compounds able to rescue the *PEX1G843D* mutation through this HTS approach may be affected by peroxisomal mosaicism and the presence of a partially functional PEX1 protein in the *PEX1G843D* mutant fibroblasts.

We reported that GSNO treatment increases peroxisome number, enhances peroxisomal protein import, and improves peroxisome function in *PEX1G843D* fibroblasts, although how GSNO accomplishes these changes remains unknown. GSNO is a peroxisomal source of NO (Corpas et al., 2017), a reactive nitrogen specie (RNS) with important roles in physiological (Desikan et al., 2004; Rosales et al., 2014; Mata-Perez et al., 2016) and pathological (Foley and O’Farrell, 2003; Tripathi et al., 2007; Fransen et al., 2012; Agurla et al., 2014; Di Cara et al., 2017) signaling pathways. NO is also produced at peroxisomes through the activity of the enzyme nitric oxide synthase (Barroso et al., 1999; Stolz et al., 2002; Loughran et al., 2005). NO has been implicated in the stress response and developmental signaling in plants (Barroso et al., 2006; Bright et al., 2009; Chaki et al., 2011; Corpas et al., 2013), while in mammals NO has been implicated in neurotransmission, immune regulation, vascular smooth muscle relaxation, and inhibition of platelet aggregation (Liu et al., 2004; Moore et al., 2009; Corti et al., 2014).

*S*-nitrosoglutathione can regulate the activities of signaling pathways by promoting the post-translational modification of key proteins in specific signaling cascades. In particular, GSNO can promote the *S*-nitrosation of proteins (Wolhuter and Eaton, 2017; Palma et al., 2020). *S*-nitrosation is a reversible post-translational modification by which NO is bound to cysteinyl residues of proteins, which can lead to either reduced or enhanced activity of a protein (Hess and Stamler, 2012; Wolhuter and Eaton, 2017). GSNO has been shown to promote the *S*-nitrosation of catalase, which modulates its activity in processes like growth, development and response to stress in plants (Corpas et al., 2019) and animals (Tyther et al., 2007), and GSNO-directed inhibition of catalase by *S*-nitrosation has been linked to obesity in humans (Palma et al., 2020). Moreover, *S*-nitrosothiols such as GSNO have been used for many years as cardiovascular therapeutics and are currently under investigation as treatments for other diseases, including Crohn’s disease (Shah et al., 2016). There, the ability of GSNO to regulate and modulate the actions of signaling cascades by post-translational modifications like *S*-nitrosation could account for its efficacy as a therapeutic for a variety of conditions.

Modulation of NO signaling has been associated with remodeling and increased biogenesis of mitochondria, Golgi, and ER in human pulmonary epithelium cells (Lee et al., 2013). Thus, GSNO could potentially increase peroxisome numbers by acting on ER or mitochondrial expansion. In our experiments, we did not observe an expansion of the ER in cells of any genotype and under any condition. However, we did observe that treatment with GSNO altered the structure of the mitochondrial network in *PEX1G843D* fibroblasts. Changes in mitochondrial shape and volume have been linked to changes in cell metabolism and signaling (McCarron et al., 2013). Interestingly, previous studies have reported that absence of functional peroxisomes in PBD-ZSS cells leads to proliferation of pleomorphic mitochondria with severely altered activity of the mitochondrial respiratory chain complexes, which is accompanied by increased production of ROS. This increased oxidative stress combined with accumulation of lipid intermediates produced by the peroxisomal ß-oxidation system was proposed to contribute significantly to the pathogenesis of PBD-ZSS (Baumgart et al., 2001). GSNO treatment of *PEX1G843D* fibroblasts rescued the mitochondrial morphology in these cells to the extent of WT fibroblasts, suggesting that GSNO improves peroxisome activity and rescues the mitochondrial morphological defects caused by alteration in peroxisome functions in *PEX1G843D* fibroblasts. Additionally, GSNO could mitigate the damage that dysfunctional peroxisomes cause to other organelles by enhancing the activity of catalase that reduces the oxidative status of peroxisome mutant cells (Fransen et al., 2012). Interestingly, *Saccharomyces cerevisiae* cells treated with GSNO exhibit enhanced catalase activity, reduced cellular oxidative stress (Lushchak and Lushchak, 2008), and less damage to organelles such as mitochondria (Fransen et al., 2017). Finally, peroxisomal NO has a defined role in the post-translational modification of proteins by *S*-nitrosation and/or nitration by which NO could modulate the activity of peroxisomal enzymes involved in ROS metabolism and affect processes like pexophagy (von Knethen and Brüne, 2002; Di Cara et al., 2017; Corpas et al., 2021). Of note, a previous study demonstrated that the observed reduction in peroxisomal protein import in *PEX1G843D* fibroblasts is not caused by defects in peroxisomal import machinery but rather by increased degradation of peroxisomes by increased autophagy (pexophagy) (Law et al., 2017). We found that compared to WT fibroblasts, *PEX1G843D* fibroblasts have a greater number of vesicles stained with LysoTracker, which stains lysosomes and late autophagosomes. Thus, GSNO could cause the increase in peroxisome number observed in our study by controlling peroxisome turnover through the regulation of pexophagy. Although a recent study showed that inhibition of pexophagy in *PEX1G843D* fibroblasts did not correlate with a reduction in cellular VLCFAs (Klouwer et al., 2021), the identification of GSNO as a pharmaceutical with the ability to improve peroxisomal function may point to yet unknown interrelationships between peroxisomal protein networks, peroxisome biogenesis, and pexophagy.

The fly *D. melanogaster* is a proven model organism for evaluating the effects of a particular drug on a human mutation (Su, 2019). Studies have shown that drugs that act on a diversity of cellular targets like the cytoskeleton, kinases, phosphatases, ion channels, cell surface receptors, chaperones, and proteases often have the same target, or act through the same mechanism, in *Drosophila* as in humans (Fernandez-Hernandez et al., 2016). Our study using a humanized *Drosophila* model for PBD-ZSS highlights the validity of a pipeline in which potential therapeutics identified by a HTS of primary cell lines are validated by administration to humanized *Drosophila*, thereby shortening the time frame for moving a therapeutic to clinical evaluation. Taken altogether our findings show that GSNO is a promising drug candidate for clinical trials in patients with mild PBD-ZSS and serves as a starting molecule for the development of other compounds optimized for the treatment of these disorders.

## Data Availability Statement

The original contributions generated for this study are included in the article/Supplementary Material, further inquiries can be directed to the corresponding authors.

## Ethics Statement

The studies involving human participants were reviewed and approved by the Office of Human Research Administration at the Kennedy Krieger Institute (Baltimore, MD, United States) to the Peroxisome Disease Laboratory, McGill University under Clinical Research Centre grants RR0052 and RR00722. Written informed consent to participate in this study was provided by the participants’ legal guardian/next of kin.

## Author Contributions

CMW performed some of the *in vivo* analyses and wrote the first draft of the manuscript. YL performed the secondary screen and analyses and the biochemical assays. YS performed the secondary screen with YL. LL performed the first trial of *in vivo* analyses. GE and AS advised on the design of the primary screen. OL and EH-C contributed to mitochondria analysis. FD designed and performed the primary and secondary screens, analyzed data from the screens, selected positive hits, and conceived and designed the experiments to characterize the hits. RR conceived the project together with AS and FD. RR and FD edited the final copy of the manuscript. All authors contributed to the article and approved the submitted version.

## Conflict of Interest

The authors declare that the research was conducted in the absence of any commercial or financial relationships that could be construed as a potential conflict of interest.

## Publisher’s Note

All claims expressed in this article are solely those of the authors and do not necessarily represent those of their affiliated organizations, or those of the publisher, the editors and the reviewers. Any product that may be evaluated in this article, or claim that may be made by its manufacturer, is not guaranteed or endorsed by the publisher.

## Footnotes

1
https://www.thebestgene.com

## Supplementary Material

Supplementary Figure 1 T6 treatment increases peroxisome number in *PEX1G843D* fibroblasts. **(A)** Detection of ABCD3-positive puncta by indirect IF microscopy in untreated and T6-treated WT fibroblasts. Scale bars, 10 μm. **(B)** Histogram reporting the quantification of ABCD3-positive puncta per cell in untreated and T6-treated WT fibroblasts. Error bars represent standard deviation (*N* = 20). Statistical significance was calculated using 2-way ANOVA. ^∗∗^*p* < 0.01. **(C)** Fluorescence images of WT fibroblasts and of *PEX1G843D* mutant fibroblasts treated with vehicle (DMSO) or T6. Green, anti-KDEL antibody staining ER. Blue, DAPI-stained nuclei. Red, phalloidin-stained cytoskeleton. Scale bars, 10 μm. **(D)** Histogram reporting the quantification of the total ER-associated fluorescence per cell in each genotype and under each condition. Error bars represent standard deviation (*N* = 20). Statistical significance was calculated using 1-way ANOVA. ns, not significant.

## Abbreviations

AAA-ATPase: ATPase associated with diverse cellular activities-ATPase
ER: endoplasmic reticulum
FA: fatty acid
FDA: Food and Drug Administration
GSNO: *S*-nitrosoglutathione
HTS: high-throughput screening
IF: immunofluorescence
LOPAC 1280: library of 1280 pharmacologically active compounds
MiNA: Mitochondrial Network Analysis
*PEX*: gene required for peroxisome assembly
PBD: peroxisome biogenesis disorder
PEX: protein encoded by a *PEX* gene
RNS: reactive nitrogen species
ROS: reactive oxygen species
VLCFA: very long-chain fatty acid.
