# Supplementary figures and images for: The Nitric Oxide Donor, S-Nitrosoglutathione, Rescues Peroxisome Number and Activity Defects in PEX1G843D Mild Zellweger Syndrome Fibroblasts
Source: Front Cell Dev Biol. 2021 Aug 9;9:714710. doi: 10.3389/fcell.2021.714710 (PMC8382563; doi:10.3389/fcell.2021.714710)

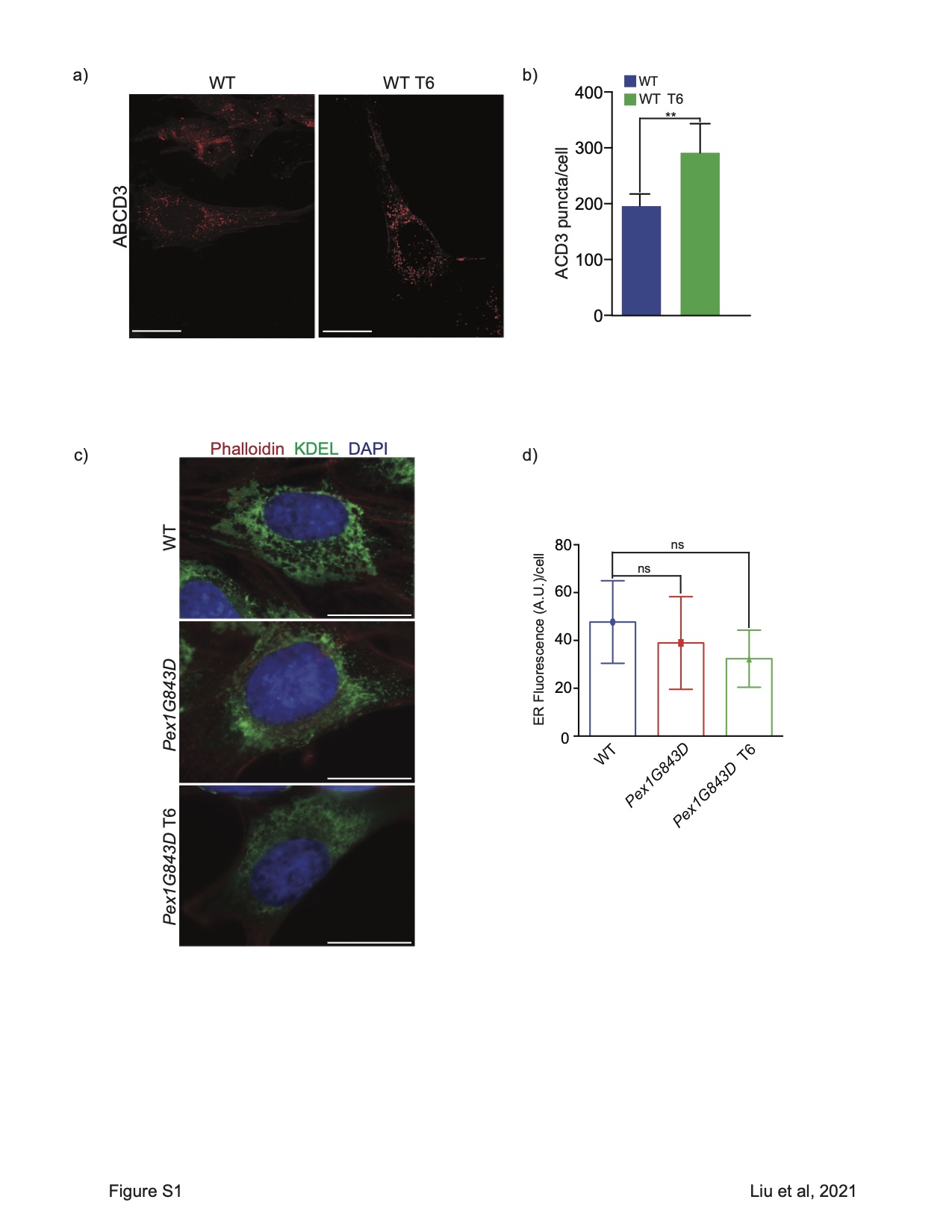

Supplement: Supplementary Figure 1 — T6 treatment increases peroxisome number in PEX1G843D fibroblasts. (A) Detection of ABCD3-positive puncta by indirect IF microscopy in untreated and T6-treated WT fibroblasts. Scale bars, 10 μm. (B) Histogram reporting the quantification of ABCD3-positive puncta per cell in untreated and T6-treated WT fibroblasts. Error bars represent standard deviation (N = 20). Statistical significance was calculated using 2-way ANOVA. ∗∗p < 0.01. (C) Fluorescence images of WT fibroblasts and of PEX1G843D mutant fibroblasts treated with vehicle (DMSO) or T6. Green, anti-KDEL antibody staining ER. Blue, DAPI-stained nuclei. Red, phalloidin-stained cytoskeleton. Scale bars, 10 μm. (D) Histogram reporting the quantification of the total ER-associated fluorescence per cell in each genotype and under each condition. Error bars represent standard deviation (N = 20). Statistical significance was calculated using 1-way ANOVA. ns, not significant. [file Image_1.JPEG]
