# Supplementary material for: The Nitric Oxide Donor, S-Nitrosoglutathione, Rescues Peroxisome Number and Activity Defects in PEX1G843D Mild Zellweger Syndrome Fibroblasts
Source: Front Cell Dev Biol. 2021 Aug 9;9:714710. doi: 10.3389/fcell.2021.714710 (PMC8382563; doi:10.3389/fcell.2021.714710)
Supplement: Supplementary file 3 [file Table_2.pdf]

Table S2. Hit compounds identified by HTS

Double-Blind ID	Sigma CAT NUM	Mol Weight	Name	Class	Action	Selectivity	Description	% Puncta Rescue
T0	N 7505	833.35	NADPH tetrasodium	Nitric Oxide	Cofactor	-	Cofactor for many enzymes, including nitric oxide synthase	40%
T1	S 1438	372.41	Sulindac sulfone	Prostaglandin	Inhibitor	-	Non-steroidal anti-inflammatory compound (NSAID); metabolite of sulindac	10%
T2	N 8534	317.22	Nilutamide	Hormone	Inhibitor	Androgen	Antiandrogen	10%
T3	R 6152	500.47	Ranolazine Dihydrochlo-ride	Lipid	Inhibitor	pFOX	pFOX (partial fatty acid oxidation) inhibitor	10%
T4	R 3277	287.32	Rutaecarpine	K+ Channel	Blocker	-	Delayed rectifier K+ channel blocker; inhibits platelet aggregation; vasoldilator	40%
T5	U-116	393.48	U-99194A	Dopamine	Antagonist	D3	D3 dopamine receptor antagonist	40%
T6	N-4146	336.32	S-Nitrosogluta-thione	Nitric Oxide	Donor	-	Nitric oxide donor in vivo	30%
T7	N 7261	299.84	Nortriptyline hydrochloride	Adrenoceptor	Inhibitor	Uptake	Tricyclic antidepressan t	20%
T8	K 1751	254.28	Ketoprofen	Prostaglandin	Inhibitor	COX-1	COX-1 selective non-steroidal anti-inflammatory (NSAID) drug	40%
T9	T7080	351.4	Tazarotene	Cell Biology	Inducer	TIG3	Tazarotene induces the expression of tazarotene-induced gene 3 (TIG3), a tumor suppressor gene	20%
T10	U-120	380.49	U0126	Phosphorylati on	Inhibitor	MEK1/MEK2	Specific inhibitor of MEK1 and MEK2 (MAP kinase kinase)	20%
